# PI3K/AKT通路在肺癌转移和耐药中的研究

**DOI:** 10.3779/j.issn.1009-3419.2011.08.10

**Published:** 2011-08-20

**Authors:** 冰晶 祝, 向东 周

**Affiliations:** 1 400038 重庆，第三军医大学研究生六队 Sixth Graduate Student Group, the Third Military University, Chongqing 400038, China; 2 400038 重庆，西南医院呼吸内科 Department of Respiratory Medicine, Southwest Hospital, Chongqing 400038, China

**Keywords:** PI3K/AKT, 信号转导, 肿瘤转移, 耐药, 肺肿瘤, PI3K/AKT, Signal transduction, Tumor metastasis, Drug resistance, Lung neoplasms

## Abstract

磷脂酰肌醇-3-激酶/丝苏氨酸蛋白激酶（phosphatidyl-inositol 3-kinase/serine-threonine kinase, PI3K/ AKT）信号通路是细胞内重要信号转导通路之一，通过影响下游多种效应分子的活化状态，在细胞内发挥抑制凋亡、促进增殖的关键作用，与人类多种肿瘤的发生发展密切相关。研究表明PI3K/AKT信号通路在恶性肿瘤细胞的增殖、血管新生和转移及对放化疗的拮抗中都起着重要作用。对PI3K/AKT信号通路的深入研究有望找到肿瘤预防和药物治疗的新靶点。本文简要介绍了PI3K/AKT信号通路的组成与功能调节，并着重阐述了其在肺癌转移和耐药中的作用。

## Abstract (en)

PI3K/AKT signal pathway is an important intracellular signal transduction pathway. It plays important roles in cell apoptosis and survival by affecting the activity of downstream effector molecules, and it is closely associated with the development and progression of human tumors. Recent researches of PI3K/AKT indicate that this pathway plays important roles in tumor cells proliferation, blood vessel neogenesis, tumor metastasis and resistance to chemotherapy and radiotherapy. The profound research of PI3K/AKT is beneficial to the prevention of tumor and to the finding of potential targets for new drugs. This article reviewed the composition, function and regulation of PI3K/AKT signal pathway, and its effect in metastasis and drug resistance of lung cancer.

肺癌是当前世界对人类健康和生命威胁最严重的肿瘤之一，其发病率和死亡率已跃居各类恶性肿瘤的前列。其致死的主要原因是转移和耐药性的出现，这是临床上治疗肺癌的难点，也是肺癌研究的热点。已有文献^[1]^报道磷脂酰肌醇-3-激酶/丝苏氨酸蛋白激酶（phosphatidylinositol 3-kinase/serine-threonine kinase, PI3K/AKT）信号通路的异常与肿瘤生长、维持和化疗耐药等方面有关。研究^[2]^表明PI3K/AKT信号通路的失调对肿瘤形成具有重要的作用，多个跨膜受体和配体结合所产生的细胞增殖信号可激活PI3K/AKT信号通路，这与肿瘤细胞的增殖和存活状态有密切关联。活化的PI3K/AKT信号通路在广泛的人类肿瘤谱中失衡，目前有研究发现肺癌转移和耐药都与PI3K/AKT信号转导通路关系密切，其主要是由于*PIK3CA*基因编码的PI3K扩增和/或其他多种因素导致的AKT过度活化^[3]^，或者是该通路某些调控成分，如类脂磷酸酶（phosphatase and tensin homolog deleted on chromosome ten, PTEN）的突变所导致的功能缺失^[4]^。因此，对PI3K/AKT信号通路的深入了解有助于找到肺癌治疗的潜在靶点。

## 1 PI3K/AKT信号通路的组成

磷脂酰肌醇3-激酶（phosphoinositide 3-kinase, PI3K）家族成员属于原癌基因，是肌醇与磷脂酰肌醇（phosphatidyl-inositol, PI）的重要激酶，是细胞内重要的信号转导分子，参与调节细胞增殖、凋亡与分化等过程，使PI环上的3’羟基磷酸化^[5, 6]^。PI3K可分为3种类型，即Ⅰ型、Ⅱ型和Ⅲ型。Ⅰ型PI3K以PI、磷脂酰肌醇-4-磷酸（phosphatidyl-inositol 4-phosphate, PIP）及磷脂酰肌醇-4, 5-二磷酸（phosphatidyl-inositol 4, 5-biphosphate, PIP2）为底物，使底物的肌醇环第3位发生磷酸化；Ⅱ型PI3K在羧基末端有C2结构域，主要磷酸化PI和PIP；Ⅲ型PI3K由催化亚基Vps34和调节亚基p150构成，以PI为底物，主要参与调控细胞生长与存活。目前研究最广泛的是能被细胞表面受体活化的Ⅰ型PI3K。Ⅰ型PI3K又分为IA和IB两个亚型，分别从酪氨酸蛋白激酶偶联受体和G蛋白偶联受体传递信号^[7]^。PI3K可被受体酪氨酸激酶和非受体酪氨酸激酶活化，在细胞膜上生成PIP3。PIP3与细胞内磷酸肌醇依赖性蛋白激酶1（3-phosphoinositide-dependent protein kinase-1, PDK1）和信号蛋白分子AKT（又名蛋白激酶B）结合，从而活化AKT。

AKT是一种丝氨酸/苏氨酸激酶，约由480个氨基酸残基组成，与蛋白激酶A（protein kinase A, PKA）和蛋白激酶C（protein kinase C, PKC）高度同源，是PI3K主要下游效应分子之一，可通过直接磷酸化多种转录因子如NF-κB和哺乳类动物雷帕霉素（the mammalian target of rapamycin, mTOR）等参与调节多种生命活动过程^[8]^。AKT分子氨基酸组成由N端到C端依次为PH结构域、中心催化结构域和短羧基端调节结构域^[9-11]^。PH结构域约含100个氨基酸残基，介导AKT活化后的膜转位过程；催化结构域含ATP结合位点，结构域内部Thr308的磷酸化为AKT活化所必需；C端调节域富含脯氨酸，并有AKT活化所必需的另一个磷酸化位点Ser473。目前发现AKT家族成员有3种亚型，包括AKT1/PKBα、AKT2/PKBβ和AKT3/ PKBγ，分别由3个不同基因编码，但蛋白质高级结构基本相同，在各种组织中均广泛表达。

## 2 PI3K/AKT信号通路的调控

PI3K/AKT信号转导通路受多因子调节，参与调节的分子主要是负反馈分子，包括PTEN、CTMP（carboxylterminal modulator protein）、PHLPP（PH domain leucinerich repeat protein phosphatase）和SHP2（Src homology phosphotyrosyl phosphatase 2）等抑癌蛋白。PTEN可拮抗PI3K/AKT信号通路，催化PIP3去磷酸化生成PIP2，从而拮抗AKT的活性^[12, 13]^。在多种肿瘤组织中均能检测到PTEN的缺失或突变，PI3K信号通路则相应显现高活性。PHLPP能特异的使AKT在Ser473位点去磷酸化，从而负调控AKT的活性^[14]^。

许多对PI3K正性调节的研究集中于其上游的活化过程，包括生长因子在内的各种刺激能依赖受体/非受体酪氨酸激酶来激活PI3K^[15-17]^。胞浆PIP3浓度升高，作为第二信使与AKT的PH结构域结合，使AKT从细胞质转位到细胞膜以及构象改变发生磷酸化而被激活。活化的AKT再进一步激活其下游分子，如mTOR、Bcl-2家族、转录因子E2F-1、糖原合成酶激酶-3（glycogen synthase kinase 3, GSK3）和S6蛋白激酶等，从而对细胞增殖、凋亡和血管生成等进行调节（图 1）。但PI3K通路并非AKT激活的惟一途径，有研究^[18]^表明EGF能促进细胞内Ca^2+^释放，增加胞浆内Ca^2+^水平，钙调蛋白是通过直接与AKT结合来调控其活性的。

**1:**
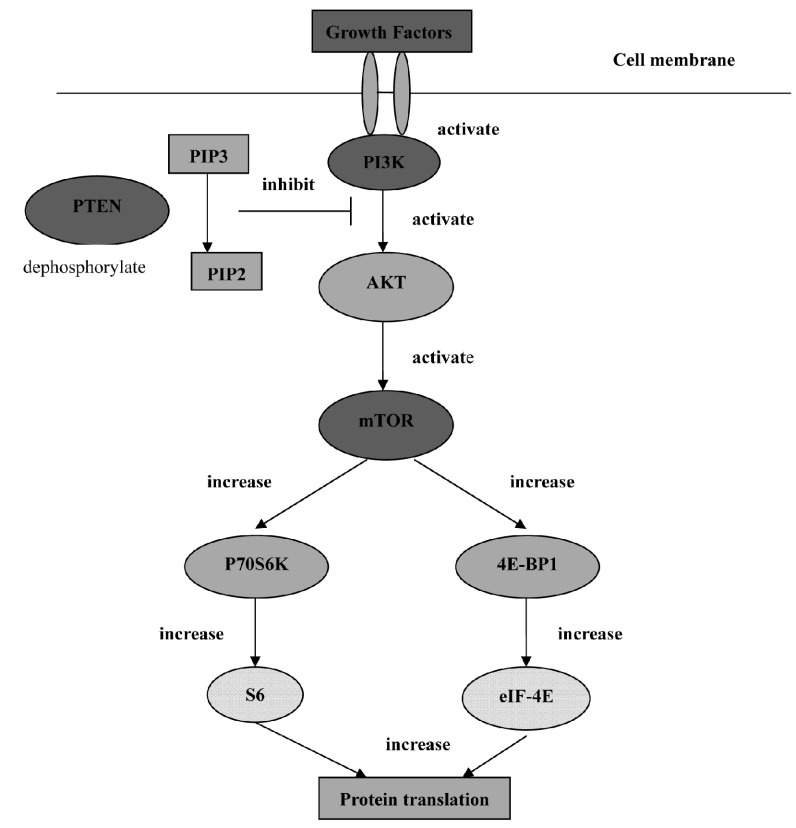
PI3K/AKT/mTOR轴^[19]^ PI3K/AKT/mTOR axis^[19]^

## 3 PI3K/AKT信号通路与肺癌的转移

恶性肿瘤的转移机制涉及肿瘤细胞遗传物质、表面结构、侵袭力、黏附能力、血管新生、淋巴管新生等多种因素。国内外研究证实PI3K/AKT信号通路与肺癌的转移密切相关。PI3K/AKT信号通路促使肺癌发生转移的机制主要有3种：①影响肿瘤黏附能力。上皮细胞-间充质细胞转换（epithelial-mesenchymal transition, EMT）可使上皮细胞获得纤维母细胞样特性，降低细胞间黏附力，增强运动能力。在细胞外信号分子与细胞膜表面的特异性受体结合后，通过细胞内不同信号转导途径活化，获得不同的转录因子，使上皮细胞表型发生不同程度的转化。有研究^[20, 21]^报道PI3K/AKT信号通路参与诱导鳞癌细胞EMT的发生：活化的PI3K产生第二信使PIP3活化下游的AKT，进而通过磷酸化作用激活或抑制下游靶蛋白，调节细胞的生存、增殖分化及细胞骨架的构成等，诱导EMT的发生，PI3K/AKT信号通路的活化增加了肿瘤细胞的侵袭性和转移性。Steelman等^[22]^认为，PI3K/AKT的持续活化及高表达与非小细胞肺癌（non-small cell lung cancer, NSCLC）发生EMT关系密切。②对肿瘤新生血管的影响。肿瘤的转移扩散依赖于新生血管形成。目前已发现多条信号通路参与调控肿瘤血管生成，PI3K/AKT是其中极重要的一条^[23]^。PI3K通过与E-Cadherin、β-Catenin、和血管内皮生长因子受体-2（vascular endothelial growth factor receptor 2, VEGFR-2）形成复合物由PI3K/AKT通路的活化参与血管内皮生长因子（vascular endothelial growth factor, VEGF）介导的内皮信号传递^[24, 25]^。PI3K/AKT信号通路还能促进肿瘤坏死因子（tumor necrosis factor, TNF）诱导的内皮细胞迁移，调节肿瘤新生血管生成^[26]^。基质金属蛋白酶（matrix metalloproteinases, MMPs）和环氧化酶-2（cyclooxygenase-2, COX-2）也能影响肿瘤新生血管的形成。在肿瘤的侵袭转移中，血小板源性生长因子（plateletderived growth factor, PDGF）经过由PI3K介导的信号通路诱导MMPs表达^[27]^，上调抗凋亡蛋白Bcl-2和激活PI3K/AKT信号通路是COX-2刺激内皮细胞血管生成的主要机制^[28, 29]^。由此可见，PI3K/AKT信号通路参与了多种因子介导的肿瘤新生血管的生成。③通过PI3K/AKT/ mTOR/p70s6k途径，促进肌动蛋白的细丝重构，促进肿瘤细胞运动转移^[30]^。

## 4 PI3K/AKT信号通路与肺癌耐药

NSCLC约占肺癌总发病率的85%，放疗、化疗是其主要治疗手段，但肿瘤细胞对化疗药物耐受性的产生是导致肺癌化疗失败，患者生存率低的主要原因之一。有研究^[31]^表明目前认为以铂类为主的联合化疗在NSCLC的治疗中有效率为50%左右，被推荐为治疗NSCLC的标准方案，不仅可减轻症状、提高生存质量，还可延长生存期。顺铂（cis-dichlorodiamine platinum, CDDP）是肺癌化疗中有效且广泛应用的一线药物，但是由于肺癌细胞耐药性问题的存在，其疗效不尽人意。CDDP等化疗药物不仅能够直接杀伤肿瘤细胞，还可以通过诱导肿瘤细胞发生凋亡而发挥作用^[32]^。抑制凋亡是肿瘤细胞产生耐药性的共同途径，细胞进入凋亡程序的能力可直接影响化疗药物发挥作用的效应。肿瘤细胞通过抑制进入凋亡程序表现出对化疗药物的耐受性，是肿瘤细胞的自我保护功能之一。选择性诱导肿瘤细胞发生凋亡已经成为肿瘤治疗的一条有效途径而倍受关注，并可能明显改善患者的预后^[33]^。PI3K/AKT信号转导通路能够调节细胞生存、增殖和分化等多种功能，是最主要的抑制细胞凋亡的信号途径^[34, 35]^。Liu等^[36]^研究证实，PI3K/AKT信号通路与肺癌细胞对CDDP的耐药密切相关，通过抑制AKT1的表达可有效逆转肺癌细胞对CDDP的耐药，采用PI3K的抑制剂LY294002能明显抑制AKT1的表达并增强肺癌耐药细胞对CDDP的敏感性。

PI3K/AKT信号转导通路抑制细胞凋亡的机制为：①通过磷酸化forkhead家族转录因子阻止其发挥调节相关基因的转录功能^[37]^；②活化转录因子NF-κB，NF-κB进入细胞核后，促进细胞增殖，抑制细胞凋亡，从而诱导抗凋亡基因*Bcl-2*、*Bcl-XL*的表达；③活化的AKT直接磷酸化cAMP应答元件结合蛋白（cAMP response element binding protein, CREB）的Ser^133^位点，诱导*Bcl-2*、*Bcl-XL*等相关基因的表达；④PI3K依赖的AKT激活，使促细胞凋亡基因*Bad*与Bcl-2或Bcl-xL解聚，Bad再与抗凋亡结合蛋白14-3-3相结合，而游离的Bcl-2发挥抗凋亡作用^[38]^；⑤抑制天冬氨酸特异性半胱氨酸蛋白酶家族成员的活化，活化的AKT可以直接催化磷酸化caspase-9的Ser196和caspase-3，使其失活从而抑制caspase导致的细胞凋亡；⑥抑制肿瘤细胞膜上TRAIL受体的活性，从而抑制由凋亡受体家族介导的细胞凋亡^[39]^；⑦通过调节细胞周期影响细胞增殖，mTOR是AKT下游的一个重要作用靶点，能够被AKT磷酸化激活，调控相关mRNA的转录，进而调节蛋白质的合成，影响细胞的增殖，AKT1诱导肺癌细胞对CDDP的耐药是通过mTOR-P70核糖体S6激酶（P70S6K1）信号途径进行^[36]^。

肿瘤分子的靶向治疗研究发现信号转导通路与肿瘤进展以及放、化疗抵抗密切相关。PI3K/AKT信号通路在肺癌细胞耐药中发挥了非常重要的作用^[36]^。研究^[40, 41]^表明PI3K/AKT的抑制剂在体外可抑制肿瘤细胞的生长，诱导肿瘤细胞进入凋亡程序，对于AKT高表达的NSCLC，使用PI3K/AKT信号通路的抑制剂能够增强化疗诱导的癌细胞凋亡，减少化疗抵抗，抑制PI3K/AKT信号通路能够有效地提高药物诱导的肺癌细胞凋亡。

## 5 PI3K/AKT信号通路抑制剂的研究

PI3K/AKT信号通路在肺癌发生、发展至关重要的众多细胞生物学过程都发挥了重要作用，抑制该信号通路成为了肺癌预防和靶向治疗的热点。PI3K/AKT信号通路的各个激酶，均有多种抑制剂处于临床前、临床研究及应用阶段。目前已经发现了该信号通路中多种激酶的小分子抑制剂（small molecule inhibitors, SMIs），其中Wortmannin和LY294002是两种广泛应用的PI3K/AKT信号通路的抑制剂。Wortmannin可与PI3K相对分子质量1.1×105催化亚基结合，特异性抑制PI3K，从而抑制PI3K/AKT信号通路。PX-866和PWT-458是近年新发现的Wortmannin的衍生物，可高效抑制PI3K。在肺癌模型中PX-866与顺铂或放疗联合使用能增强抗肿瘤作用^[42]^。此外，PWT-458还能增强紫杉醇抗肿瘤的疗效^[43]^。LY294002可完全特异性的抑制PI3K的活性，能和PI3K竞争性结合ATP位点，抑制AKT的活性。LY294002与常规放化疗联合应用具有协同作用，并可以减少不良反应，对标准化方案产生耐药的肿瘤可考虑联合应用PI3K抑制剂^[44]^。吴等^[45]^发现LY294002可抑制A549细胞增殖，且随LY294002浓度的增大及作用时间的延长，生长抑制率也随之增大；同时LY294002可抑制裸鼠移植瘤的生长，使移植瘤的磷酸化AKT蛋白表达下降，提示LY294002可通过抑制PI3K活性，进而调控AKT表达及活化，从而抑制肺癌细胞的生长。研究^[46-48]^表明Wortmannin和LY294002均能通过抑制AKT磷酸化进而发挥抗肿瘤的效应。

PI3K抑制剂在NSCLC的研究中证明在体内单独使用及与化疗药物联合应用均能增加肿瘤细胞的凋亡^[49]^。磷酸化的AKT通过将信号传递给众多下游分子参与肿瘤发生发展，是目前肿瘤靶向研究的一个热点。抑制AKT的活性能抑制下游靶基因转录，从而抑制下游信号传递。目前研究的AKT抑制剂主要有两种形式：脂类抑制剂和通过高通量筛选化合物库所得的SMIs。脂类抑制剂有perifosine、磷脂酰肌醇烷脂和PX-316。对于perifosine的研究最为透彻，能抑制AKT的膜转位，并能通过降低AKT的活性抑制多种肿瘤细胞生长^[50]^。SMIs主要有API-2、API-59CJ-Ome和吲哚-嘧啶A-443654等。

## 6 结语

细胞生存信号的转导对肺癌的发生发展非常重要，而PI3K/AKT信号通路被认为是癌细胞存活的首要通路。目前对PI3K/AKT信号通路的作用及其具体调节机制认识尚不完全，许多问题还有待进一步探讨。随着对PI3K/ AKT信号转导通路及其在肺癌中重要作用研究的逐渐深入，针对该信号通路开发新的肿瘤治疗药物对肺癌的治疗具有十分重要的意义。

近年以PI3K/AKT信号通路分子作为靶点的抗肿瘤药物已得到很好的开发，这些AKT特异性抗肿瘤药物可拮抗AKT抗凋亡作用，破坏癌细胞的耐药性，诱导癌细胞发生凋亡^[51]^。此外，还能有效提高化疗药物顺铂、紫杉醇对体外肺癌细胞抑制作用的敏感性，抑制PI3K/AKT信号通路可明显提高肺癌的化疗效果，减少化疗药物的剂量，提高患者对治疗的依从性。但是特异性抑制剂因其毒性较强使临床应用具有一定限制性，此外，在基础研究及动物模型取得的抗癌效果对于肺癌病例是否具有足够的效力而改变癌症的进展及PI3K/AKT信号通路的下游底物并未完全了解，还有待进一步研究。PI3K/AKT信号通路的任何一个环节的异常都与肺癌的发生发展密切相关。因此，全面、系统地深入研究PI3K/AKT信号转导通路分子机制对以信号通路作为靶点的肺癌防治工作具有非常重要的意义。
